# Therapeutic Inhibition of Cathepsin S Reduces Inflammation and Mucus Plugging in Adult *β*ENaC-Tg Mice

**DOI:** 10.1155/2021/6682657

**Published:** 2021-03-19

**Authors:** Ryan Brown, Donna M. Small, Declan F. Doherty, Leslie Holsinger, Robert Booth, Richard Williams, Rebecca J. Ingram, J. Stuart Elborn, Marcus A. Mall, Clifford C. Taggart, Sinéad Weldon

**Affiliations:** ^1^Airway Innate Immunity Research (AiiR) Group, Wellcome-Wolfson Institute for Experimental Medicine, School of Medicine, Dentistry and Biomedical Sciences, Queen's University Belfast, Belfast, UK; ^2^ViroBay Inc., Menlo Park, CA, USA; ^3^Patrick G Johnston Centre for Cancer Research, School of Medicine, Dentistry and Biomedical Sciences, Queen's University Belfast, Belfast, UK; ^4^Wellcome-Wolfson Institute for Experimental Medicine, School of Medicine, Dentistry and Biomedical Sciences, Queen's University Belfast, Belfast, UK; ^5^Department of Translational Pulmonology, Translational Lung Research Center Heidelberg (TLRC), German Center for Lung Research (DZL), University of Heidelberg, Heidelberg, Germany; ^6^Department of Pediatric Pulmonology, Immunology and Critical Care Medicine, Charité-Universitätsmedizin Berlin, Berlin, Germany; ^7^Berlin Institute of Health (BIH), Berlin, Germany

## Abstract

**Background:**

Elevated levels of the cysteine protease cathepsin S (CatS) are associated with chronic mucoobstructive lung diseases such as cystic fibrosis (CF) and chronic obstructive pulmonary disease (COPD). We have previously demonstrated that prophylactic treatment with a CatS inhibitor from birth reduces inflammation, mucus plugging, and lung tissue damage in juvenile *β*-epithelial Na+ channel-overexpressing transgenic (*β*ENaC-Tg) mice with chronic inflammatory mucoobstructive lung disease. In this study, we build upon this work to examine the effects of therapeutic intervention with a CatS inhibitor in adult *β*ENaC-Tg mice with established disease.

**Methods:**

*β*ENaC-Tg mice and wild-type (WT) littermates were treated with a CatS inhibitor from 4 to 6 weeks of age, and CatS^−/−^*β*ENaC-Tg mice were analysed at 6 weeks of age. Bronchoalveolar lavage (BAL) fluid inflammatory cell counts were quantified, and lung tissue destruction and mucus obstruction were analysed histologically.

**Results:**

At 6 weeks of age, *β*ENaC-Tg mice developed significant airway inflammation, lung tissue damage, and mucus plugging when compared to WT mice. CatS^−/−^*β*ENaC-Tg mice and *β*ENaC-Tg mice receiving inhibitor had significantly reduced airway mononuclear and polymorphonuclear (PMN) cell counts as well as mucus plugging. However, in contrast to CatS^−/−^*β*ENaC-Tg mice, therapeutic inhibition of CatS in *β*ENaC-Tg mice had no effect on established emphysema-like lung tissue damage.

**Conclusions:**

These results suggest that while early CatS targeting may be required to prevent the onset and progression of lung tissue damage, therapeutic CatS targeting effectively inhibited airway inflammation and mucus obstruction. These results indicate the important role CatS may play in the pathogenesis and progression of mucoobstructive lung disease.

## 1. Background

Cathepsin S (CatS) is a cysteine protease predominantly expressed in antigen-presenting cells where it plays a crucial role in the processing of major histocompatibility complex class II (MHCII) [1]. While other proteases in this family require an acidic environment for optimum activity, CatS is active at a more neutral pH allowing it to maintain its activity in the extracellular environment [2] and thereby contribute to disease pathogenesis via the degradation of important antimicrobial proteins and antiproteases [3–6] as well as through direct tissue damage as a result of its potent elastolytic [7] and collagenolytic [8] activity. Respiratory disease is a major global health concern accounting for 10% of all disability-adjusted life years (DALYs), and 5 of these diseases, including chronic obstructive pulmonary disease (COPD), are among the most common causes of death worldwide [9]. The multifaceted roles of CatS in the pathogenesis of various airway diseases such as COPD and cystic fibrosis (CF) have been extensively reviewed elsewhere [10–14].

Doherty et al. recently demonstrated that a protein phosphatase 2A–CatS pathway plays a significant role in the development of airspace enlargement and loss of lung function in a cigarette smoke-induced murine model of COPD [15]. CatS expression and activity were shown to be significantly higher in the lung tissue of current smokers (both non-COPD and COPD) compared with those in never-smokers and correlated positively with smoking history [16]. In addition, exposure of primary human bronchial epithelial cells to cigarette smoke extract triggered the activation of the P2X7 receptor, which in turn drives CatS upregulation [16]. In the context of CF lung disease, elevated CatS levels and activity have previously been shown in the lungs of adults and children with the disease [17, 18]. Pulmonary inflammation and mucus obstruction are overlapping features of CF and COPD lung disease [19–22]. In addition, the development of emphysema is a common feature of COPD and has also been described in CF [21, 23]. Emphysema is characterised by damage to the alveolar air sacs leading to airspace enlargement which reduces the efficiency of oxygen exchange [24, 25]. Emphysema is irreversible, and there are presently few effective therapeutic options available for this condition, which is particularly prevalent in patients with COPD [26].

We have previously demonstrated that inhibiting CatS from birth in an *in vivo* model of CF-like lung disease reduces pulmonary inflammation, airway mucus obstruction, and lung tissue destruction via a mechanism that is mediated, in part, via protease activated receptor-2 [27]. However, whether this beneficial effect can be achieved when treatment is started after the onset of disease is unknown. In this study, the phenotypic effects of therapeutic administration of a CatS inhibitor in the *β*ENaC-Tg mouse model of chronic mucoobstructive lung disease and *β*ENaC-Tg mice ablated for the CatS gene (CatS^−/−^*β*ENaC-Tg) were analysed to elucidate whether sustained knockdown or early intervention with an inhibitor is needed to dampen airway inflammation and prevent lung tissue damage and airway mucus plugging. To achieve this goal, we determined the effects of genetic ablation of CatS in adult (6 weeks old) *β*ENaC-Tg mice and compared these to age-matched adult *β*ENaC-Tg mice that had received CatS inhibitor for 2 weeks from 4 weeks of age.

## 2. Materials and Methods

### 2.1. Experimental Animals and Inhibitor Treatment

All mice were housed in specific pathogen-free (SPF) facilities where housing and experimentation were carried out in accordance with the Animal (Scientific Procedures) Act 1986 and current guidelines approved by the Queen's University Belfast Ethical Review Committee. The animals were maintained on a 12 hr cycle of light followed by 12 hr cycle of darkness with free access to chow and water. *β*ENaC-Tg mice [28] were backcrossed on to the C57BL/6 background as previously described [29]. CatS^−/−^ mice [30] were intercrossed with *β*ENaC-Tg mice for 5 generations to generate the double mutant CatS^−/−^*β*ENaC-Tg mice as previously described [27]. *In vivo* CatS inhibition was achieved using VBY-999 (Virobay Inc., Menlo Park, CA) [27, 31] or I.6 (QUB) [32, 33]. VBY-999 and I.6 are reversible CatS inhibitors that bind and block the active site of CatS. VBY-999 (100 mg/kg) or vehicle control (5% dextrose, Sigma-Aldrich, Dorset, UK) was delivered subcutaneously once per day for 14 days, and mice were sacrificed 12 hours after the final treatment. Dosing was based on advice from the manufacturer and previously published data [27, 31].

### 2.2. *In Vivo* Bacterial Infection and Inhibitor Treatment

*Pseudomonas aeruginosa* (PA01) or *Staphylococcus aureus* (QO51) was grown in nutrient broth (VWR, Leuven, Belgium) overnight before being refreshed for 3 hr and diluted to OD600 = 0.3 (*P. aeruginosa*) or OD600 = 5 (*S. aureus*) in sterile PBS. *β*ENaC-Tg mice (3 weeks old) received a subcutaneous injection of I.6 CatS inhibitor (200 mg/kg) or vehicle control (4% DMSO in peanut oil, Sigma-Aldrich). After 20 min, mice received an intranasal delivery of 20 *μ*l of either *P. aeruginosa* (OD 0.3), *S. aureus* (OD 5) or PBS control under isoflurane anaesthetic. Mice were sacrificed 6 hr later, and bronchoalveolar lavage (BAL) fluid collected and processed as described below. Lungs and spleens were collected and homogenised in sterile PBS. Samples were plated on cetrimide (Sigma-Aldrich) (*P. aeruginosa*) or mannitol (VWR, Leuven, Belgium) (*S. aureus*) agar for quantification of CFU.

### 2.3. Bronchoalveolar Lavage Fluid Collection and Cell Counts

Mice were sacrificed by means of an intraperitoneal injection of pentobarbital (Pentoject, Animalcare, York, UK). The lungs were lavaged, and the cell-free BAL fluid was stored at -80°C as previously described [27]. Total BAL fluid cell counts were determined using a haemocytometer (Brightline, Harsham, UK) and 0.4% trypan blue (Sigma-Aldrich) to exclude dead cells. Differential cell counts were performed from cytospins (Cytospin 2, Thermo-Shandon, Cheshire, UK) stained with May-Grünwald Giemsa (Merck, Darmstadt, Germany), with a total of at least 400 cells counted per mouse imaged using a Leica DM55 brightfield microscope (Leica Microsystems, UK).

### 2.4. Lung Histology and Airway Morphology

Histological and morphometric analyses were performed as previously described [27, 28, 34, 35]. Lung tissue sections were cut at 6 *μ*m and stained with Alcian blue-periodic acid Schiff (AB-PAS) (Alfa Aesar, Ward Hill, MA) for airway mucus content or haematoxylin and eosin (H&E) (Leica Biosystems Ltd., Newcastle, UK) for mean linear intercept (MLI) and destructive index (DI). Airway mucus content was assessed in the left proximal main axial airways by determining percentage of the airway containing AB-PAS-positive material using ImageJ software. MLI (a measure of distal airspace enlargement) and DI (a measure of alveolar wall destruction) analyses were performed on five randomly selected frames per mouse at 200x power and analysed using ImageJ. MLI was determined by means of a 3-line counting grid [36]. The number of alveolar walls intercepting the 3-line grid was counted, and the average number of intercepts per line was calculated. This value was divided by the length of the grid line to give average air space diameter. DI was determined by means of a 42-point grid [35]. Structures lying under these points were classified as normal or destroyed alveolar and/or duct spaces. Points falling over other structures, such as duct walls and alveolar walls, or points falling over positions where the entire structure was not visible were not included in the calculation. Alveolar or duct spaces were classed as normal if surrounded by intact walls or by walls disrupted in only one place. Alveolar spaces were classed as destroyed if the wall of the alveolus was disrupted in 2 or more places or there were 2 or more disruptions of contiguous alveoli that were part of the structures opening onto a single duct system. Duct spaces were classed as destroyed if 2 or more isolated islands of lung parenchyma were observed in the lumen of a duct. Both alveolar and duct spaces were classed as destroyed if the structure was lined by cuboidal epithelium, but was clearly not a normal airway, with or without obvious breaks in the walls or a classic emphysematous lesion was present. DI was calculated as

(1) $$\begin{array}{c} \left(\frac{D}{D+N}\right)\times 100, \end{array}$$

*D* = Destroyed alveolar/duct space.

*N* = Normal alveolar/duct space.

### 2.5. Protein Extraction and Quantification

Lungs were harvested from mice and immediately snap frozen in liquid nitrogen. Frozen lungs were homogenised in 400 *μ*l RIPA buffer supplemented with 100X Halt™ Protease and Phosphatase Inhibitor Cocktail (Thermo Fisher Scientific, Horsham, UK). Homogenates were incubated on ice for 15 min before centrifuging at 17,000 x g for 10 min. Supernatants were collected and stored at -80°C until further use. Protein in lysates were quantified by Bradford assay. Briefly, 2 *μ*l of sample or standard was added to a 96-well plate. A 200 *μ*l aliquot of Bradford reagent (Thermo Fisher Scientific) was added to each well, and absorbance was measured immediately at 595 nm (Biotek Synergy HT plate reader). Sample values were measured against the standard curve to determine protein concentration.

### 2.6. Western Blotting

Denatured lung lysate or BAL fluid samples were separated on 12% SDS-PAGE gels and transferred onto nitrocellulose membrane (GE Healthcare, Buckinghamshire, UK). Membranes were blocked and incubated with anti-CD74 (BD Biosciences, Oxford, UK), anti-CatS (R&D Systems, Abingdon, UK), and *γ*-tubulin (Sigma-Aldrich) overnight at 4°C. Membranes were washed, incubated with HRP-conjugated secondary antibodies, developed using chemiluminescent substrate (Western Lightning, PerkinElmer, Coventry, UK), and viewed using Syngene G:Box and GeneSnap software (Syngene, Cambridge, UK).

### 2.7. Cathepsin S Activity Assay

CatS activity in BAL fluid from 6-week-old WT and *β*ENaC-Tg was determined using the substrate Z-Phe-Arg-7-amido-4-methylcoumarin, Hydrochloride (FR-AMC; Merck) as previously described [27].

### 2.8. Enzyme-Linked Immunosorbent Assay (ELISA)

Levels of KC and IL-13 in BAL fluid were quantified by ELISA as per manufacturer's instructions (R&D Systems).

### 2.9. Statistics

All data were analysed using GraphPad Prism 8 (GraphPad Software Inc., San Diego, CA) and are reported as mean ± SEM. Statistical analysis were performed using unpaired two-tailed *t*-test or one-way ANOVA with Tukey's multiple comparisons test for normally distributed data and two-tailed Mann-Whitney test or Kruskal-Wallis test for nonnormally distributed data as appropriate. *P* < 0.05 was accepted to indicate statistical significance; ^∗^*P* < 0.05, ^∗∗^*P* < 0.01, ^∗∗∗^*P* < 0.001, ^∗∗∗∗^*P* < 0.0001.

## 3. Results

### 3.1. CatS Is Upregulated in the Lungs of Adult *β*ENaC-Tg Mice with Chronic Mucoobstructive Lung Disease

**Figure 1:**
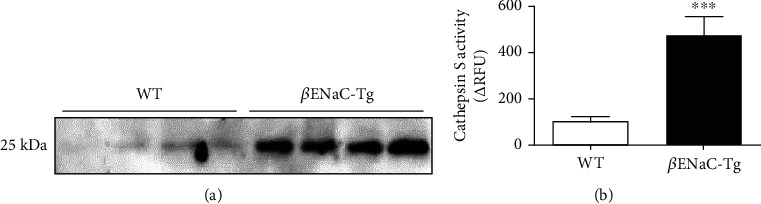
Levels and activity of cathepsin S are upregulated in the lungs of adult *β*ENaC-Tg mice. (a) Western blot of cathepsin S in BAL fluid from adult (6 weeks old) *β*ENaC-Tg and WT mice (*n* = 4 per group). Cathepsin S activity in (b) BAL fluid from adult WT and *β*ENaC-Tg mice (*n* = 8 per group) was determined using FR-AMC substrate (pH 7.5). Results expressed as change (Δ) in relative fluorescence units (*Δ*RFU) over time. ^∗∗∗^*P* < 0.001 (unpaired two-tailed *t*-test).

We have previously demonstrated upregulation of CatS protein and activity in BAL fluid from 2-week-old juvenile *β*ENaC-Tg mice [27]. To determine if CatS is also upregulated in the lungs of adult *β*ENaC-Tg mice, we compared BAL fluid CatS protein levels and activity from WT and *β*ENaC-Tg mice at 6 weeks of age. We observed increased CatS protein (Figure 1(a)) and activity (Figure 1(b)) in *β*ENaC-Tg mice when compared to WT controls. These results validate the use of adult *β*ENaC-Tg mice in this study to compare genetic ablation of CatS and late therapeutic CatS inhibition in the context of established chronic lung disease.

### 3.2. Genetic Knockout of CatS Prevents Airway Inflammation

**Figure 2:**
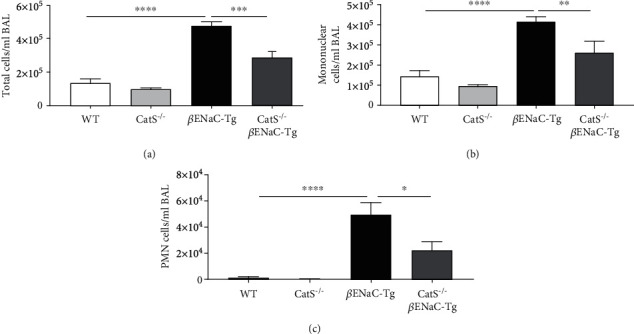
Cathepsin S knockdown from birth prevents airway inflammation in adult *β*ENaC-Tg mice. (a) Total cell, (b) mononuclear, and (c) polymorphonuclear (PMN) counts were quantified in BAL fluid from 6-week-old WT, CatS^−/−^, *β*ENaC-Tg, and CatS^−/−^*β*ENaC-Tg mice (*n* = 5‐12 per group). ^∗^*P* < 0.05, ^∗∗^*P* < 0.01, ^∗∗∗^*P* < 0.001, ^∗∗∗∗^*P* < 0.0001 (one-way ANOVA with Tukey's multiple comparisons test).

To examine the effects of genetic ablation of CatS on airway inflammation in adult *β*ENaC-Tg mice, we compared inflammatory cell counts in BAL fluid from WT, CatS^−/−^, *β*ENaC-Tg, and CatS^−/−^*β*ENaC-Tg mice at 6 weeks of age. As expected, *β*ENaC-Tg mice had increased BAL total cell counts compared to WT controls (Figure 2(a)) associated with increased mononuclear (Figure 2(b)) and polymorphonuclear (PMN) (Figure 2(c)) cell counts. Knockout of CatS significantly decreased total cell counts in BAL fluid from *β*ENaC-Tg mice but had no effect on WT mice (Figure 2(a)). This observed decrease in total inflammatory cells in CatS^−/−^*β*ENaC-Tg compared to *β*ENaC-Tg mice was associated with decreases in airway mononuclear and PMN cell counts (Figures 2(b) and 2(c)).

### 3.3. Genetic Knockout of CatS Prevents the Development of Mucus Obstruction and Emphysema-Like Lung Damage

**Figure 3:**
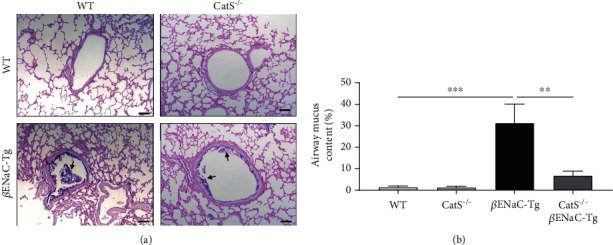
Cathepsin S knockdown from birth prevents mucus obstruction. (a) Representative images of adult WT, CatS^−/−^, *β*ENaC-Tg, and CatS^−/−^*β*ENaC-Tg mouse lung sections stained with Alcian blue-periodic acid Schiff. Black arrows indicate airway mucus, scale bar = 100 *μ*m. (b) Airway mucus quantification is expressed as the percentage of the total airway area containing mucus (*n* = 6‐7 group). ^∗∗^*P* < 0.01, ^∗∗∗^*P* < 0.001 (one-way ANOVA with Tukey's multiple comparisons test).

**Figure 4:**
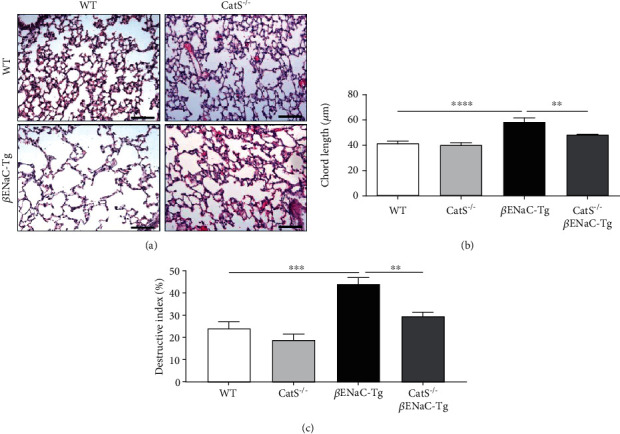
Cathepsin S knockdown from birth limits lung tissue destruction. (a) Representative images of lung sections stained with haematoxylin and eosin (H&E) used to assess airway damage. Scale bar = 100 *μ*m. (b) Mean linear intercept and (c) destructive index measurements were quantified from H&E-stained lung sections as detailed in materials and methods (*n* = 5‐6 per group). ^∗∗^*P* < 0.01, ^∗∗∗^*P* < 0.001, ^∗∗∗∗^*P* < 0.0001 (one-way ANOVA with Tukey's multiple comparisons test).

Genetic knockout of CatS prevented the development of both airway mucus obstruction and lung tissue damage in 2-week-old juvenile *β*ENaC-Tg mice [27]. To determine if genetic ablation of CatS results in a sustained prevention of airway mucus plugging and lung tissue destruction through to 6 weeks of age, we compared airway mucus content and lung damage in WT, CatS^−/−^, *β*ENaC-Tg, and CatS^−/−^*β*ENaC-Tg mice at 6 weeks of age (Figure 3). As expected from previous studies [27, 34, 37], adult *β*ENaC-Tg mice displayed significant airway mucus plugging compared to WT, which was significantly decreased in CatS^−/−^*β*ENaC-Tg mice (Figures 3(a) and 3(b)). MLI and DI were increased in *β*ENaC-Tg compared to WT mice (Figures 4(a)–4(c)). No differences were observed between CatS^−/−^ and WT mice. However, knockout of CatS in adult *β*ENaC-Tg mice resulted in a significant reduction in both MLI and DI (Figures 4(a)–4(c)).

### 3.4. Pharmacological Inhibition of CatS Reduces Airway Inflammation

**Figure 5:**
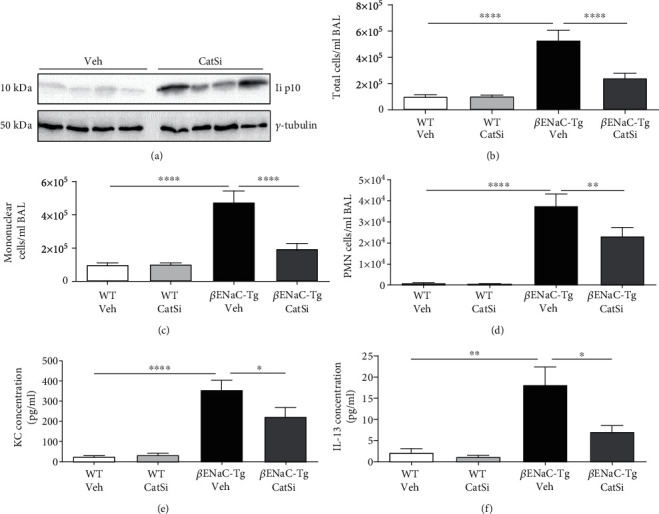
Pharmacological targeting of cathepsin S reduces airway inflammation in adult *β*ENaC-Tg mice. Four-week-old WT and *β*ENaC-Tg mice were treated daily with cathepsin S inhibitor VBY-999 (CatSi; 100 mg/kg) or dextrose vehicle control (veh) for 14 days. (a) Inhibition of cathepsin S in *β*ENaC-Tg mice alters invariant chain (Ii) processing and results in the accumulation of the 10 kDa intermediate (p10), which was detected in lung lysate by Western blot (*n* = 4 per group). BAL fluid (b) total cell, (c) mononuclear, and (d) polymorphonuclear (PMN) cell counts were quantified (*n* = 8‐20 mice per group). BAL fluid levels of (e) KC and (f) IL-13 were quantified by ELISA (*n* = 6‐11 mice per group). ^∗∗^*P* < 0.01; ^∗∗∗∗^*P* < 0.0001 (one-way ANOVA with Tukey's multiple comparisons test).

Prophylactic CatS inhibitor treatment from birth significantly inhibited inflammatory cell recruitment in the lungs of 2-week-old *β*ENaC-Tg mice [27]. To determine if pharmacological inhibition of CatS has therapeutic effects when started in adult *β*ENaC-Tg mice with established lung disease, we treated 4-week-old mice with CatS inhibitor for 2 weeks in this study. To confirm the inhibition of CatS, we examined the invariant chain (Ii) fragment p10 by Western blotting. CatS is involved in the processing of the Ii, and inhibition of CatS results in the accumulation of a 10 kDa fragment, Ii p10 [1, 30]. Accumulation of Ii p10 was observed in mice receiving inhibitor compared to vehicle control (Figure 5(a)) confirming CatS inhibition. This late therapeutic intervention regimen leads to significantly reduced inflammatory cell infiltration in *β*ENaC-Tg mice compared to vehicle controls (Figure 5(b)). Specifically, we observed significant reductions in airway mononuclear and PMN cell counts (Figures 5(c) and 5(d)). In addition, levels of the chemokine KC and the type 2 cytokine IL-13 were significantly reduced in CatS inhibitor-treated *β*ENaC-Tg mice (Figures 5(e) and 5(f)). Given the proinflammatory actions of CatS and its role in antigen presentation [1, 38], the potential for increased pulmonary infection risk in CatS inhibitor-treated *β*ENaC-Tg mice was investigated. However, the anti-inflammatory effects associated with pharmacological CatS inhibition did not significantly alter the course of acute bacterial infection in *β*ENaC-Tg mice (Supplemental Figure 1).

### 3.5. Pharmacological Inhibition of CatS Reduces Airway Mucus Plugging but Does Not Revert Emphysema-Like Lung Damage

**Figure 6:**
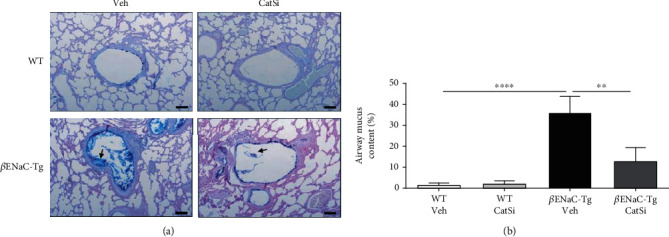
Pharmacological targeting of cathepsin S reduces airway mucus plugging. Four-week-old WT and *β*ENaC-Tg mice were treated daily with cathepsin S inhibitor VBY-999 (CatSi, 100 mg/kg) or dextrose vehicle control (veh) for 14 days. (a) Representative images of adult WT and *β*ENaC-Tg mice lung sections stained with Alcian blue-periodic acid Schiff. Black arrows indicate airway mucus, scale bar = 100 *μ*m. (b) Airway mucus quantification is expressed as the percentage of the total airway area containing mucus (*n* = 8‐9 per group). ^∗∗^*P* < 0.01, ^∗∗∗∗^*P* < 0.0001 (one-way ANOVA with Tukey's multiple comparisons test).

**Figure 7:**
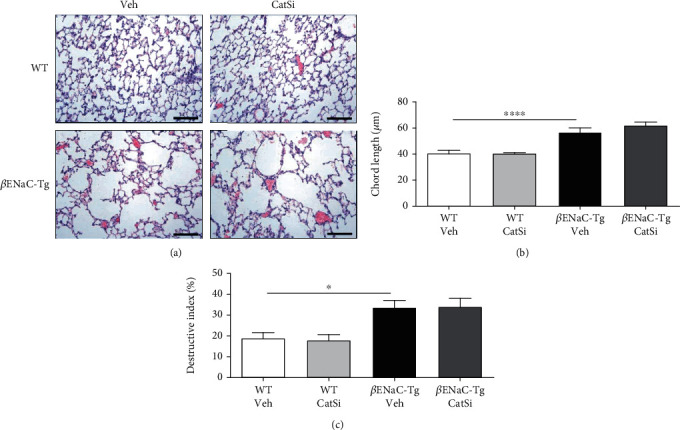
Cathepsin S inhibition does not reduce emphysema-like lung damage. Four-week-old WT and *β*ENaC-Tg mice were treated daily with cathepsin S inhibitor VBY-999 (CatSi, 100 mg/kg) or dextrose vehicle control (veh) for 14 days. (a) Representative images of lung sections stained with haematoxylin and eosin (H&E) used to assess airway damage. Scale bar = 100 *μ*m. (b) Mean linear intercept and (c) destructive index measurements were assessed from the H&E-stained lung sections as detailed in materials and methods (*n* = 7‐8 per group). ^∗^*P* < 0.05, ^∗∗∗∗^*P* < 0.0001, (b) one-way ANOVA with Tukey's multiple comparisons test, (c) Kruskal-Wallis test with Dunn's multiple comparisons test.

Next, we determined the effects of late therapeutic CatS inhibitor intervention on airway mucus plugging. We started treatment of WT and *β*ENaC-Tg mice at 4 weeks of age at which stage mucus plugs will have formed in the airways. As previously described [28], the airways from *β*ENaC-Tg mice contained mucus plugs that obstructed the airway (Figure 6(a)). However, pharmacological targeting of CatS significantly reduced the area of the airways obstructed by mucus in *β*ENaC-Tg mice (Figures 6(a) and 6(b)). To determine if delayed therapeutic intervention with a CatS inhibitor could also reduce lung tissue damage, MLI and DI were quantified in 6-week-old WT and *β*ENaC-Tg mice (Figure 7). MLI (Figure 7(b)) and DI (Figure 7(c)) were significantly increased in *β*ENaC-Tg mice compared to WT controls. However, inhibition of CatS had no effect on MLI or DI in either WT or *β*ENaC-Tg mice. These results indicate that late intervention with a CatS inhibitor does not revert established emphysema-like lung damage in this model of mucoobstructive lung disease.

## 4. Discussion

In this study, we used adult *β*ENaC-Tg mice as a model of established chronic mucoobstructive lung disease [28, 34, 39]. *β*ENaC-Tg mice provide a useful model to test potential therapeutic strategies targeting inflammation, mucus plugging, and tissue damage in the lungs. We have demonstrated increased CatS activity and protein levels in the lungs of both juvenile [27] and adult *β*ENaC-Tg mice. Similar to our *in vivo* findings, the lungs of patients with CF and COPD display elevated CatS, adding to the burden of proof that elevated CatS is a feature of the chronically mucoobstructed lung [10, 16, 27, 40, 41]. Data from this study demonstrate that the protective effects of genetic ablation of CatS on the development of airway inflammation, mucus plugging, and lung tissue damage are sustained in adult *β*ENaC-Tg mice. In addition, delayed pharmacological inhibition of CatS in adult *β*ENaC-Tg mice significantly reduced the well-known hallmarks of chronic airway inflammation and mucus obstruction. However, late treatment with a CatS inhibitor had no effect on the progression of lung tissue damage, suggesting that early pharmacological intervention may be crucial to prevent this process from occurring.

In CF, evidence of lung remodelling has been detected in children with CF, and may be associated with the early protease-antiprotease imbalance that characterises the CF lung [12, 42–44]. Our previous work identified CatS as a potential key mediator of early CF lung disease pathogenesis, and *in vivo* prophylactic targeting of CatS from birth resulted in significant reductions in neutrophilia, mucus obstruction, and lung remodelling in juvenile *β*ENaC-Tg mice [18, 27]. It has also been demonstrated that chronic exposure of CatS^−/−^ mice to cigarette smoke results in reduced inflammation and lung damage compared to smoke-exposed WT littermates, confirming a role for CatS in the inflammatory burden and airspace enlargement observed in smoking-induced lung disease [15]. These findings are in agreement with early studies in which pharmacological pretreatment with a CatS inhibitor and genetic knockout of CatS prevented IFN*γ*-induced emphysema [45]. CatS inhibition did not significantly alter the course of acute bacterial infection in *β*ENaC-Tg mice. We hypothesise that the low numbers of lymphocytes in *β*ENaC-Tg mice suggests an ensuing immune response to infection that may not be affected by reductions in MHCII loading as a result of CatS inhibition [28, 34]. Additionally, inactivation of a number of antimicrobial peptides, including surfactant protein A and LL-37, by CatS has been observed [5, 6]. Therefore, improved host antimicrobial defence may also help balance the host response and prevent an exacerbated respiratory infection in CatS inhibitor-treated mice.

The lack of efficacy of delayed CatS inhibition on lung tissue damage may be due to the progressive damage that develops during the first 4 weeks of life before the initiation of CatS inhibitor treatment [34]. Previous studies have demonstrated that *β*ENaC-Tg mice present with early onset distal airspace enlargement with significant increases in MLI observed from as early as 3 days of age onwards [34, 46]. Additionally, increased airway inflammation, as measured by BAL fluid total inflammatory cell counts, is observed in juvenile compared to adult *β*ENaC-Tg mice [34]. Taken together, these data highlight the significant lung tissue damage occurring during the first weeks of life before onset of treatment with the CatS inhibitor in this study. Furthermore, the lack of efficacy of CatS inhibition in preventing lung tissue damage in adult *β*ENaC-Tg mice may occur as a result of changes in the protease populations present in adult versus juvenile mice. In this context, both neutrophil elastase (NE) and matrix metalloproteinase (MMP)-12 are upregulated on the surface of neutrophils and macrophages, respectively, in adult *β*ENaC-Tg mice [47, 48]. Using genetic knockouts, distinct roles for these proteases have been identified. In contrast to CatS, NE was found to contribute to airway neutrophilia and structural lung damage but not mucus obstruction, whereas MMP-12 was identified as a key player in emphysema formation but not leukocyte recruitment or airway mucus obstruction [47, 48]. In addition, IL-1 signalling has been implicated in structural lung damage in the *β*ENaC-Tg model. Both genetic deletion and pharmacological targeting of the IL-1 receptor with anakinra reduced structural lung damage in *β*ENaC-Tg mice [37, 49]. Further studies analysing the involvement and interplay of other proteases, e.g., NE, MMP-12, as well as the IL-1 signalling pathway in *β*ENaC-Tg mice with CatS knockdown will expand our understanding of the pathogenesis of the lung phenotype associated with this important mouse model.

Previous studies in the *β*ENaC-Tg mouse model testing other therapeutic interventions have also shown variations in the efficacy of late versus preventative therapeutic treatment. In a study by Zhou et al., preventative treatment with amiloride, a specific ENaC channel blocker administered to inhibit Na^+^ hyperabsorption and airway surface dehydration that trigger mucoobstructive lung disease in this model [28, 50] reduced airway inflammation and mucus plugging [51]. However, this effect was lost when initiation of treatment was delayed until after the onset of lung disease [51]. The irreversible lung damage observed in adult *β*ENaC-Tg mice is a typical feature of chronic lung diseases such as COPD and one which so far remains incurable. Although CatS inhibitor treatment in adult *β*ENaC-Tg mice did not lead to reparative or regenerative changes in the lung, early intervention with a CatS inhibitor may have the potential to alter the course of structural lung disease as indicated by data from CatS^−/−^ mouse models [15, 27, 45].

## 5. Conclusion

In summary, therapeutic pharmacological inhibition of CatS in adult *β*ENaC-Tg mice significantly reduced the well-known hallmarks of chronic airway inflammation and mucus obstruction. However, in contrast to the protective effects of genetic ablation of CatS, late treatment with a CatS inhibitor had no effect on the progression of lung tissue damage in adult mice, suggesting that early pharmacological intervention may be crucial to prevent this process from occurring. This novel data may have important implications for the development of therapeutics in the future aimed at limiting the progression of inflammatory lung disease in patients.

## Data Availability

The datasets used or analysed during the current study are available from the corresponding author on reasonable request.

## Abbreviations

BAL: Bronchoalveolar lavage
CatS: Cathepsin S
CF: Cystic fibrosis
COPD: Chronic obstructive pulmonary disease
DI: Destructive index
ENaC: Epithelial sodium channel
MLI: Mean linear intercept.

## Supplementary Materials

Supplementary Materials Supplemental Figure 1: inhibition of cathepsin S has no effect on bacterial clearance or associated airway inflammation in *β*ENaC-Tg mice.
