# Supplementary material for: Therapeutic Inhibition of Cathepsin S Reduces Inflammation and Mucus Plugging in Adult βENaC-Tg Mice
Source: Mediators Inflamm. 2021 Mar 19;2021:6682657. doi: 10.1155/2021/6682657 (PMC8004367; doi:10.1155/2021/6682657)
Supplement: Supplementary Materials — Supplemental Figure 1: inhibition of cathepsin S has no effect on bacterial clearance or associated airway inflammation in βENaC-Tg mice. [file 6682657.f1.docx]

**SUPPLEMENTAL RESULTS**

**SUPPLEMENTAL FIGURE LEGEND**

**Supplemental Figure 1. Inhibition of cathepsin S has no effect on bacterial clearance or associated airway inflammation in βENaC-Tg mice.**

βENaC-Tg mice were infected with (A-E) *Staphylococcus aureus* (n = 7-10 per group) or (F-I) *Pseudomonas aeruginosa* (n = 5 per group) and treated with a single subcutaneous dose of cathepsin S inhibitor I.6 (CatSi, 200 mg/kg) or peanut oil vehicle control (veh). BAL fluid (A,F) Total cell, (B,G) mononuclear and (C,H) polymorphonuclear (PMN) cell counts were quantified. (D, I) BAL fluid and (E, J) lung CFU were quantified from cetrimide (*P. aeruginosa*) or mannitol (*S. aureus*) agar plates. (A-D, F-H, J) unpaired two-tailed t test, (E, I) two-tailed Mann-Whitney test.
